# Lockdown Social Isolation and Lockdown Stress During the COVID-19 Pandemic in China: The Impact of Mindfulness

**DOI:** 10.3389/fpsyg.2022.778402

**Published:** 2022-04-27

**Authors:** Jianfeng Li, Luyang Zhou, Beatrice Van der Heijden, Shengxiao Li, Hong Tao, Zhiwen Guo

**Affiliations:** ^1^Department of Big Data Management and Application, School of Business, Hubei University, Wuhan, China; ^2^Department of Economics and Management, Yuanpei College, Shaoxing University, Shaoxing, China; ^3^Department of Business Administration, School of Business, Shaoxing University, Shaoxing, China; ^4^Institute for Management Research, Radboud University, Nijmegen, Netherlands; ^5^Faculty of Management, Open University of the Netherlands, Heerlen, Netherlands; ^6^Department of Marketing, Innovation and Organisation, Ghent University, Ghent, Belgium; ^7^School of Business, Hubei University, Wuhan, China; ^8^Kingston Business School, Kingston University, London, United Kingdom; ^9^Department of Human Resource Management, School of Business, Hubei University, Wuhan, China

**Keywords:** lockdown social isolation, lockdown job insecurity, lockdown financial insecurity, lockdown stress, mindfulness, COVID-19

## Abstract

This study is aimed to examine the impact of mindfulness in the relationship between social isolation, job and financial insecurity, and stress during the lockdown period of the COVID-19 pandemic. Drawing on Conservation of Resources theory, Psychological Contract theory, Mindfulness theory, and Awareness notion, we propose that lockdown job insecurity partially mediates the link from lockdown social isolation to lockdown financial insecurity, and that the relationship between lockdown social isolation and lockdown stress is mediated as follows: first, simple partial mediation through both lockdown job and financial insecurity and second, sequential mediation through lockdown job and financial insecurity, respectively. Moreover, we assume that mindfulness moderates the relationship between lockdown financial insecurity and lockdown stress. The results from our SEM analyses, using a sample of 1,356 respondents in China, support all the research hypotheses. Based on this empirical work, this study concludes that mindfulness, which is considered by many people to play a role in reducing stress during the COVID-19 lockdown period, is de facto endangering their mental health (that is, they experience more stress) instead. Theoretical and practical implications, as well as limitations and proposals for future research are discussed.

## Introduction

Lockdown social isolation related to the COVID-19 (Corona Virus Disease-2019) pandemic may not only lead to financial loss, because of not being able to work, but also to post-traumatic stress symptoms (Brooks et al., 2020; Grabowski et al., 2021; Owens et al., 2022; World Health Organization (WHO), 2022). Due to the lockdown social isolation, the unemployment rate has increased sharply, and daily working hours have declined quickly around the world (e.g., Cai et al., 2021; Coibion et al., 2020; Internal Labour Organization (ILO), 2021; Lee et al., 2020). Therefore, many employees have led their life in anxiety, distress or depression related to the job and financial insecurity they have experienced (Ganson et al., 2020; Gasparro et al., 2020; Nanda, 2020; Wang et al., 2020; Wilson et al., 2020; Basyouni and El Keshky, 2021; Cai et al., 2021). As a possible means to cope with the stress and anxiety that is entailed by the pandemic lockdown, many researchers focused on mindfulness practice (e.g., Weis et al., 2021; Zhu et al., 2021; Matta et al., 2022). Mindfulness practice has already been widely used to treat chronic pain, anxiety, skin diseases, relapse of depression, insomnia, substance abuse, alcohol dependence, eating disorder, heart disease and cancer (e.g., Kabat-Zinn, 2015; Baer et al., 2019; Burrowes et al., 2022). Indeed, at the beginning of the outbreak of the COVID-19 pandemic, the Chinese National Health Commission recommended people to practice mindfulness meditation to cope with the lockdown stress.

However, previous studies also revealed that mindfulness may have negative effects on one’s mental health (e.g., stress and anxiety) under high levels of awareness and uncontrolled settings (Sahdra et al., 2017; Britton, 2019; Farias et al., 2020; Matta et al., 2022; Xu et al., 2022). In particular, in contrast to the mainstream view that mindfulness practice always implies self-relaxing and low physiological arousal, some researchers found that mindfulness meditation might bring about physiological arousal as well (e.g., Lumma et al., 2015). Therefore, more empirical insight is needed to better understand the mechanisms behind the relationship between lockdown social isolation and lockdown stress, and whether and how mindfulness can play a role in this regard. So far, it is not clear whether mindfulness has a positive or negative impact on employees’ perceived stress during the period of the lockdown and social isolation. Hence, this study aims to examine whether and how lockdown social isolation has an impact on employees’ perceptions of job and financial insecurity and their experienced lockdown stress, and to investigate how mindfulness plays a role in these relationships within a Chinese context.

The current research will make three contributions. First, based on the theory of Conservation of Resources (COR, cf. Hobfoll, 1989, 2001), this study sheds more light on the possible role of lockdown social isolation as a predictor for lockdown job insecurity and its consequences, herewith offering a psychological perspective to understand and capture the impact of the COVID-19 pandemic. Given the lack of research on studying the antecedents of job insecurity (Lee et al., 2018; Lin et al., 2021), this empirical study extends previous academic work and adds to the knowledge in this field with a Chinese sample. Second, based on COR theory (ibid.) and Psychological Contract theory (Ashford et al., 1989; Keim et al., 2014), by examining the indirect effects of lockdown social isolation on lockdown stress, via lockdown job and financial insecurity, we provide empirical insights into these relationships. Third, drawing on Mindfulness theory (Kabat-Zinn, 2015) and Awareness notion (Kernis and Goldman, 2004), by exploring the role of mindfulness as a predictor for lockdown stress and as a moderator in the relationship between lockdown financial security and lockdown stress, our findings indicate that mindfulness can not only reduce employees’ perceived stress, but also increase their experienced levels of stress. These findings are important, not only for employees’ welfare but also for organizational and societal sustainability in future potential crises.

## Theoretical Background and Hypotheses

### Lockdown Social Isolation and Lockdown Stress

Social isolation can be defined as “an objective state marked by few or infrequent social contacts” (Holt-Lunstad, 2020, p. 1). Recent research has shown that after acute social isolation individuals generally feel lonely and crave for social contacts (Tomova et al., 2020). Social contacts are among the most fundamental elements in human beings’ lives. Accordingly, social isolation may result in adverse effects on people’s well-being (Tuzovic and Kabadayi, 2020). Unfortunately, in order to reduce person-to-person transmission of COVID-19, many countries adopted lockdown and social distancing polices, and many employees were forced to work at home or were suspended from work, which might result in lockdown stress related to this social isolation (e.g., lack of face-to-face interaction with colleagues and others).

Stress usually refers to “the experience of opportunities or threats that people perceive as important and also perceive they might not be able to handle or deal with effectively” (George and Jones, 2006, p. 275), and perceived lockdown stress occurs when one feels his or her work and life to be threatened by lockdown and social distancing. Earlier studies have revealed that stress could exert an adverse impact, not only on one’s physiological health but also on one’s psychological well-being (George and Jones, 2006; Yaribeygi et al., 2017).

We posit that the relationship between lockdown social isolation and lockdown stress can be interpreted by COR theory (Hobfoll, 1989, 2001). More specifically, Hobfoll (ibid.) suggested that resources include objects, personality traits, conditions, and energies, and that an individual has a motive to strive to obtain, retain, and protect the resources he or she values, and minimizes any threats of resource losses. For this reason, somebody may experience stress when they (1) perceive any threats of resource losses, (2) actually lose resources, and (3) are unable to obtain a new resource after investment of resources (ibid.).

During the COVID-19 pandemic, employees might need to apply both physiological and psychological resources for coping with the consequences of social isolation, due to the lockdown and social distancing policies, which might lead to resource losses, both at work and in their private life. Recent research also found that during the COVID-19 pandemic, social isolation is a stressor in itself (Van Bavel et al., 2020; Rudolph et al., 2021), and that employees’ perceived social isolation, produced by the lack of face-to-face connectedness with colleagues, has a positive impact on their stress levels (Toscano and Zappalà, 2020). In addition, social isolation related to COVID-19 was found to be linked to traumatic stress (Boyraz et al., 2020). Based on the outline given above, we argue that social isolation generated by the lockdown and social distancing might lead to an increase in employees’ perceived lockdown stress. Therefore, we propose the following hypothesis:

*Hypothesis 1*: Lockdown social isolation has a positive impact on lockdown stress during the COVID-19 lockdown period.

### The Mediating Roles of Lockdown Job Insecurity and Lockdown Financial Insecurity

The lockdown and social distancing produced by the COVID-19 pandemic have resulted in a global economic recession (Lambert et al., 2020). Previous research has discovered that during the economic declines, employees might experience more job insecurity generated by a reduction in their working hours and income, and poor physical and mental health (Frone, 2018). Job insecurity refers to “perceived powerlessness to maintain desired continuity in a threatened job situation” (Greenhalgh and Rosenblatt, 1984, p. 438). In line with Mohr’s (2000) four-phase model of job insecurity, in the present study, perceived lockdown job insecurity could be defined as employees’ perceptions regarding the likelihood of losing their job due to the lockdown and social distancing during the COVID-19 pandemic, which reflects a state of public awareness and acute job insecurity at an individual level. Under this circumstance, job insecurity is viewed as a stressor (De Witte et al., 2016), and it could bring adverse effects to employees during the COVID-19 pandemic (Ganson et al., 2020; Aguiar-Quintana et al., 2021).

Building on Keim et al. (2014) scholarly work, we use Psychological Contract theory to understand the relationship between lockdown social isolation and job insecurity in this study. Psychological contracts refer to the expectations of the employee-employer relationship beyond the formal contract of employment (Smithson and Lewis, 2000). Previous research has already shown that sound psychological contracts could guarantee employees’ fair benefits and wage income and give them a sense of control, which could further reduce their perceived job insecurity (Ashford et al., 1989; Keim et al., 2014).

In particular, Ashford et al. (1989) suggested that any factors that threaten this sense of control could elicit a sense of job insecurity, and, as such, function as its antecedents. According to Sverke and Hellgren’s (2002) model of job insecurity, both the objective situation (i.e., labor market characteristics, organizational change, employment contact, and uncertain future for the organization) and subjective characteristics (i.e., perceived employability, perceived control, family responsibility, and need for security) are considered as potential predictors of job insecurity. Existing research has also illustrated that external environment threats (e.g., high national unemployment rate related to the COVID-19 pandemic) could lead to employees’ perceived job insecurity, because the lack of control related to uncertainty could result in a sense of job insecurity (Keim et al., 2014; Shoss, 2017; Spurk and Straub, 2020) and a series of issues related to job insecurity (e.g., fear, emotional exhaustion) (Baert et al., 2020; Chen and Eyoun, 2021).

Due to worrying about the uncertainty of future economic conditions in the COVID-19 pandemic, many employees may concurrently feel insecure about their continuous financial capacity. After all, in practice, the social isolation due to the lockdown not only restricts people’s working patterns, but also reduces their amount of working hours. Indeed, a recent study found that 21.1% of the participants feared losing their job, and that 49.9% feared a reduction in their wages and benefits in the COVID-19 crisis (Baert et al., 2020). In China, as of November 2020, an unemployed person has been jobless for 7 months (211 days) on average, and 51% of the unemployed have been jobless for more than half a year (Cai et al., 2021). In addition, during this period of unemployment, their main income sources came from their family members’ support (47.5%) and from their own savings (38.1%) (ibid.). Therefore, in this context, lockdown social isolation related to the COVID-19 pandemic may act as a predictor of employees’ perceived job insecurity due to both the objective situation such as a high unemployment rate and an uncertain future for the organization, and due to subjective characteristics such as low perceived control over situation and high family responsibility.

In summary, lockdown social isolation related to the COVID-19 pandemic may do damage to the employee-employer psychological contract, which potentially may induce employees’ job insecurity and further trigger their experienced financial insecurity. Accordingly, we propose the following hypothesis:

*Hypothesis 2*: The relationship between lockdown social isolation and lockdown financial insecurity during the COVID-19 lockdown period is partially mediated by lockdown job insecurity.

As important stressors, job and financial insecurity may affect employees’ perceived lockdown stress. According to COR theory (Hobfoll, 1989, 2001), employees may feel stressed when they experience threats of resource losses and are unable to gain any resources to compensate for this (Hobfoll, 1989). As the loss of employment in the COVID-19 pandemic period can give rise to a significant decline in employees’ mental health and well-being (Cai et al., 2021), employees’ perceived job insecurity related to the lockdown and social distancing may serve as a potential threat of resource losses (Ismail, 2015), and therefore intensify their perceived lockdown stress. Indeed, some researchers have found that during the COVID-19 pandemic period, social isolation may bring about both work-related financial losses and post-traumatic stress symptoms (Brooks et al., 2020), and that job insecurity was positively associated with anxiety and depression (Ganson et al., 2020; Gasparro et al., 2020; Wilson et al., 2020; Aguiar-Quintana et al., 2021).

In China, a recent study also found that the lockdown measure slowed down the progress of returning to work for the unemployed population, and increased the possibility of a worsening employment market (Cai et al., 2021). Correspondingly, lockdown social isolation due to the COVID-19 pandemic may trigger the perception of job insecurity and financial loss, and further incite employees to invest more time and energy at work in order to maintain their present positions and relevant earnings. As a result, in response to the threat of losing their job and the accompanying financial loss, employees have to consume their reserved resources, which may intensify their perceived levels of stress. Thus, we propose the following hypotheses:

*Hypothesis 3*: The relationship between lockdown social isolation and lockdown stress in the COVID-19 lockdown period is indirect and mediated in the following ways:
H3a: Simple partial mediation through lockdown job insecurity—lockdown social isolation positively affects lockdown job insecurity, and lockdown job insecurity positively affects lockdown stress;
H3b: Simple partial mediation through lockdown financial insecurity—lockdown social isolation positively affects lockdown financial insecurity, and lockdown financial insecurity positively affects lockdown stress;
H3c: Sequential mediation through lockdown job insecurity and lockdown financial insecurity—lockdown social isolation positively affects lockdown job insecurity, lockdown job insecurity positively affects lockdown financial insecurity, and lockdown financial insecurity positively affects lockdown stress.

### The Moderating Role of Mindfulness

Mindfulness is defined as “paying attention in a particular way: on purpose, in the present moment, and nonjudgmentally” (Kabat-Zinn, 2015, p. 3), and it is also considered as “an enhanced attention to and awareness of current experience or present reality” (Brown and Ryan, 2003, p. 822). Open or receptive awareness and attention are viewed as core characteristics of mindfulness (e.g., Martin, 1997). Kabat-Zinn (2015) believed that as this process of paying attention on purpose, in the present moment, and nonjudgmentally could make them to be more conscious, sober and receptive to the present reality. The latter implies that individuals learn how to live in harmony with their own stress and pain through mindfulness practice.

Psychological acceptance and living in the present are the most important mechanism of mindfulness in coping with stress and pain. On the one hand, by allowing the existence of negative emotions and ideas, rather than escaping, struggling or falling into them, individuals could alleviate the negative experiences, to a great extent, and herewith buffer their perceived stress levels. On the other hand, by paying attention to the present moment, individuals could lay down the regret for the past and the anxiety about the future. For this reason, mindfulness practice could result in a change in individuals’ experienced stress and pain levels, and herewith change their life (Kabat-Zinn, 2015). In essence, this means that individuals only continue in the present moment after having gone through a previous moment, and that they should pay attention to the present moment only, and wholeheartedly participate in each and every moment, before moving through the next one. As such, those employees with high levels of mindfulness in the pandemic crisis might just observe anxiety and depression of perceived job and financial insecurity, due to the Covid-19 pandemic, without judging or evaluating them, and therefore further live in harmony with these insecurities with receptive awareness.

In fact, mindfulness is considered to be an individual internal resource (Ni et al., 2021), which can help employees to deal with the lockdown stress. As mindfulness involves attention to current experiences and events, rather than to past or future ones, and receptive awareness, it can facilitate individuals to observe internal and external stimuli without judging or evaluating them, and therefore raise their awareness of other resources. Correspondingly, according to COR theory (Hobfoll, 1989, 2001), mindfulness can help employees to accept their current level of resources, depend less on available resources in their surroundings, and enhance their awareness of alternative resources (see also Kroon et al., 2015). In addition, as a loss of resources may result in feelings of stress and burnout (Hobfoll, 1989, 2001), employees with a high level of mindfulness may strive to maintain their level of resources more actively.

Indeed, recent empirical studies have confirmed that mindfulness practice is an effective means of coping with COVID-19-related stress and anxiety (Weis et al., 2021; Zhu et al., 2021). Therefore, in the context of our empirical study, we posit that one can handle their perceived lockdown stress more effectively by improving their level of mindfulness through well-thought out mindfulness practice. Thus, we propose the following hypothesis:

*Hypothesis 4*: The mindfulness of employees is negatively associated with their perceived lockdown stress in the COVID-19 lockdown period.

Although in scholarly research in this field, mindfulness practice is normally regarded as a simple and efficient way to reduce stress (e.g., Weis et al., 2021; Zhu et al., 2021), it sometimes may make things even worse. For example, in their review study, Baer et al. (2019) found that many participants felt more depressed, anxious, and had less self-esteem after mindfulness meditation. In a similar vein, Farias et al. (2020), in their meta-analysis, discovered that the total pooled prevalence of meditation adverse events was 8.3%, and that the most common symptoms reported were stress (20%), cognitive anomalies (25%), depression (27%), and anxiety (33%). In addition, Lumma et al. (2015) found that core meditations in improving meta-cognitive skills and compassion are related to physiological arousal, which may induce negative psychological responses such as stress and pain.

Kabat-Zinn (2015) thought that mindfulness exercise could drive an individual to face their emotions, such as pain, grief, anger and fear (all being emotions that one usually does not want to face soberly and express consciously, in the deepest of their hearts). The power of mindfulness lies in its specific practice and application, and it can enable individuals to grasp their selves more comprehensively through systematic self-observation, self-exploration and conscious behavior, and, through this, mindfulness practice can liberate the mind and the soul (Kabat-Zinn, 2015). The key to mindfulness practice is to develop a receptive and non-judgmental attitude toward self-awareness in the present moment. If individuals ignore the present moment, and are driven by deep-seated fear and insecurity, actions and behaviors in an unconscious way, this will inevitably do harm for their health and well-being (ibid.).

In China, only a few well-known enterprises such as Alibaba, Huawei and Didi have successively offered mindfulness courses to their employees and managers in recent years^1^, and very few employees across the nation have regularly learned how to relieve their stress levels by mindfulness practice during the lockdown period. Accordingly, the vast majority of Chinese employees might not pay attention on purpose and nonjudgmentally in the present moment. On the contrary, in light of Kernis and Goldman’s (2004) Awareness notion that a high level of awareness often means potential costs (e.g., pain and stress), people might be confined to the awareness of lockdown social isolation experience itself, and the financial insecurity that it entails, and thus perceive high lockdown stress. More specifically, the authors suggested that a high awareness score implies that an individual has more knowledge of their internal states, such as affect, motives, goals, and self-relevant cognition. Such self-knowledge may lead to potential costs. In particular, (1) some forms of self-knowledge may be painful, (2) certain emotions per se may be unsettling and threatening, (3) self-reflection may itself induce unpleasant affect, and (4) the awareness of one’s multifaceted self-concept may intensify role strain (ibid.). Accordingly, if one only holds much self-knowledge of their internal states, one may experience negative effects. In other words, the more aware an individual feels, the more painful and stressful he or she may feel.

Concretely, the more aware employees feel about their financial insecurity in the COVID-19 lockdown period, the more painful and stressful they may feel, as financial insecurity related to the COVID-19 lockdown is highly unsettling, and threatening. Indeed, in empirical work, some scholars already found that high levels of COVID-19 awareness are associated with increased anxiety and depression (Landa-Blanco et al., 2021; Liu et al., 2021). Hence, based on the above-mentioned background, and in the context of our empirical study, we propose the following hypothesis:

*Hypothesis 5*: The positive impact of lockdown financial insecurity on lockdown stress during the COVID-19 lockdown period will become stronger with the increase of the level of mindfulness.

**FIGURE 1:**
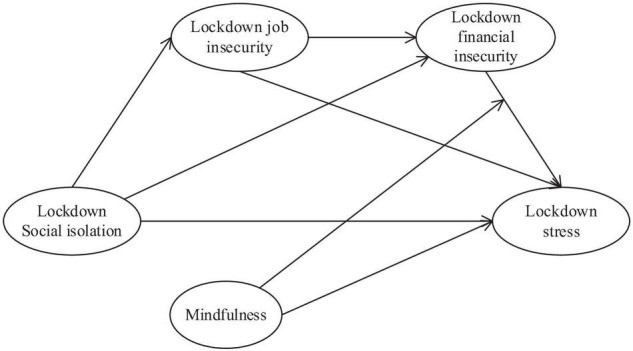
Hypothesized model.

Our study model is depicted in Figure 1.

## Methodology

### Samples and Procedure

A series of pretests of the questionnaire was conducted as a preliminary investigation of the reliability and validity of the key measures in our inquiry. Based on analyzing the outcomes using a convenience pilot sample of 319 respondents and consulting a small group of respondents for their advice on how to improve the questionnaire, we refined the formal survey for final distribution.

For the sake of the representativeness of our dataset, we employed the proportionate stratified sampling method. We partitioned the target population into three strata based on the numbers of infected COVID-19 cases across Chinese provinces by July 29, 2020. The first stratum was Hubei Province, where the COVID-19 pandemic was the most severe with the epicenter Wuhan city included. The second stratum consisted of six provinces, that is Guangdong, Henan, Zhejiang, Hunan, Anhui, and Heilongjiang, with moderate pandemic severity. The other provinces were allocated to the third stratum, where the lowest pandemic severity took place. We aimed to collect a sample of 1,500–2,000 observations, in order to control for low levels of margin of error especially when conducting structural equation modelling. We targeted approximately 20% or 300–400 observations from the first stratum, and approximately 40% or 600–800 observations from the second and third strata.

We commissioned two data service companies in China to administer the survey. While both companies were reputable, they seemed to have their own popularity in different regions of China. Our research team members also independently sent out online questionnaires through WeChat groups. All respondents participated to the survey voluntarily and anonymously, and received financial tokens that could be used toward the WeChat mobile payment social media app. A total of 2,157 online questionnaires were completed; 72% collected by data service companies, and 28% by the research team through WeChat.

We strictly validated all the filled-out questionnaires. Those respondents who were not involved in any kind of lockdown restrictions, those who were out of work (were unemployed or had retired) or worked in rural areas, those who lived outside Mainland China (i.e., Hong Kong, Macao, Taiwan or abroad), and those who reported that they worked in governmental or non-profit organizations (like hospitals and schools), were deleted from the final data set. The finally validated data set included 1,356 questionnaires (62.9%), among which 245 (18% from the total of 1,356) were derived from the first stratum, 545 (40% from the total of 1,356) were from the second stratum, and 566 (42% from the total of 1,356) were from the third stratum.

### Measures

#### Lockdown Social Isolation

Based on the COVID-19 lockdown background, the social isolation measure concentrated on external facets (Zavaleta et al., 2017), such as the infrequency of contact with social network members (Brummett et al., 2001), the low level of participation in social activities (Ellison and George, 1994; Thoits and Hewitt, 2001; Benjamins, 2004), and the socially disconnected status (Cornwell and Waite, 2009). Four items were used to assess lockdown social isolation (α = 0.82) (e.g., “I hadn’t participated in many activities which I should have been in if there had been no lockdown,” 1 = “strongly disagree,” 5 = “strongly agree”).

#### Lockdown Job Insecurity

Lockdown job insecurity was assessed using a four-item scale (α = 0.86) (e.g., “During the COVID-19 lockdown, I often felt that my current job might become unstable due to the pandemic,” 1 = “strongly disagree,” 5 = “strongly agree”), which was adapted from previous studies like Anderson and Pontusson (2007); Carr and Chung (2014), Vander Elst et al. (2014).

#### Lockdown Financial Insecurity

Lockdown financial security was assessed using a five-item scale (α = 0.90) (e.g., “During the COVID-19 lockdown, I worried that the sources of my income would be reduced due to the pandemic,” 1 = “strongly disagree,” 5 = “strongly agree”), which was adapted from previous studies like Lawrence et al. (2013), Odle-Dusseau et al. (2018), Weinstein and Stone (2018).

#### Lockdown Stress

Building on the 7-item stress subscale of Antony et al. (1998) and the 4-item stress subscale of Lee et al. (2019), and based on the results of our pretests, we dropped the ‘agitated’ and ‘intolerant’ items and created a 5-item scale to assess lockdown stress (α = 0.92). A sample item is: “During the COVID-19 lockdown, I found it hard to wind down (1 = strongly disagree, 5 = strongly agree).

#### Mindfulness

A six-item scale was used to measure mindfulness (α = 0.76) (e.g., “I can usually describe how I feel at the moment in considerable detail,” 1 = strongly disagree, 5 = strongly agree), which was adapted from the Cognitive and Affective Mindfulness Scale-Revised (CAMS-R) (Feldman et al., 2007). Concurrently, the Chinese version of CAMS-R or Ch-CAMS-R (Chan et al., 2016) was referenced.

Table A1 demonstrates all details of the measures.

### Control Variables

In our data analysis, we controlled for variables like lockdown experience, working status, family companion, sex, age, educational background, and income level. The experience of strict lockdown (i.e., the whole city or the whole region was strictly isolated to stop the spread of COVID-19) was coded as 1, and the experience of less strict lockdown or partial lockdown was coded as 0. Regarding working status, we divided the respondents into two groups: individuals who have paused working as a measure of epidemic control were coded as 1; individuals who worked in their companies or at home online were coded as 0. Family companion was coded as 1 if individuals lived with their family members, and it was coded as 0 if living alone.

We used the approach of Fair (1978) for the data coding for the other control factors. For gender, female and male were coded as 0 and 1 respectively. Age was divided into six categories, under 20, 20–29, 30–39, 40–49, 50–59, and 60 or over, which were coded as 19, 25.5, 35.5, 45.5, 55.5, and 65.5 respectively. Educational level included junior middle school graduate or below, high school graduate, junior college graduate, college graduate, Master’s degree, and Ph.D., which were coded as 9, 12, 15, 16, 19, and 22 respectively. Average month income, 0.05 = 1000 CNY or below; 0.2 = 1000–3000 CNY; 0.4 = 3000–5000 CNY; 0.75 = 5000–10,000 CNY; 1.5 = 10,000–20,000 CNY; 3.5 = 20,000–50,000 CNY; 7.5 = 50,000–100,000 CNY; 15 = over 100,000 CNY.

## Analysis and Results

### Descriptive Statistics and Correlation Analysis

Table 1 illustrates the descriptive statistics for all study variables. Lockdown social isolation significantly and positively correlated with lockdown job insecurity (*r* = 0.127, *p* < 0.01), lockdown financial insecurity (*r* = 0.175, *p* < 0.01), lockdown stress (*r* = 0.236, *p* < 0.01) and mindfulness (*r* = 0.088, *p* < 0.01). Lockdown job insecurity significantly and positively correlated with lockdown financial insecurity (*r* = 0.649, *p* < 0.01) and lockdown stress (*r* = 0.395, *p* < 0.01), while it significantly and negatively correlated with mindfulness (*r* = –0.097, *p* < 0.01). Lockdown financial insecurity significantly and positively correlated with lockdown stress (*r* = 0.468, *p* < 0.01) and significantly and negatively correlated with mindfulness (*r* = –0.093, *p* < 0.01). Mindfulness significantly and negatively correlated with lockdown stress (*r* = –0.116, *p* < 0.01).

**TABLE 1:** Mean, standard deviations, and correlations matrix for the whole sample.

	Mean	SD	Range	1	2	3	4	5	6	7	8	9	10	11	12
(1) Lockdown social isolation	3.54	0.90	1–5	1											
(2) Lockdown job insecurity	3.05	1.01	1–5	0.127**	1										
(3) Lockdown financial insecurity	3.46	1.01	1–5	0.175**	0.649**	1									
(4) Lockdown stress	2.80	1.07	1–5	0.236**	0.395**	0.468**	1								
(5) Mindfulness	22.97	3.32	11–30	0.088**	–0.097**	–0.093**	–0.116**	1							
(6) Experience of the lockdown	0.31	0.46	0–1	0.137**	0.108**	0.106**	0.142**	–0.027	1						
(7) Working status	0.24	0.42	0–1	–0.031	0.110**	0.177**	0.062*	–0.058*	0.114**	1					
(8) Family companion	0.91	0.28	0–1	–0.071**	–0.004	0.038	0.006	0.013	–0.082**	0.064*	1				
(9) Gender	0.58	0.49	0–1	0.007	–0.024	–0.006	–0.041	0.000	–0.033	–0.012	–0.098**	1			
(10) Age	33.93	7.76	19–65.5	–0.091**	–0.179**	–0.118**	–0.100**	0.070*	–0.077**	–0.004	0.108**	0.196**	1		
(11) Educational level	15.74	1.42	9–21	0.085**	–0.053	–0.114**	–0.010	0.053	–0.030	–0.189**	–0.032	–0.059*	–0.161**	1	
(12) Income	1.56	2.36	0.05–15	–0.015	–0.021	–0.073**	–0.090**	0.060*	–0.015	–0.030	–0.013	0.042	0.031	0.132**	1

*p < 0.05, **p < 0.01. Sample size = 1,356.

The control variables were all related to one or more of the model variables (see Table 2 for specific details). The mean value of family companion, *M* = 0.91, indicated that most of the respondents stayed with their family. All in all, these outcomes provided preliminary evidence for the proposed hypotheses.

**TABLE 2:** Testing the discriminant validity of the constructs.

CFA models	*x* ^2^	*df*	*x*^2^/*df*	CFI	TLI	RMSEA	SRMR
4 factors	470.86	84	5.61	0.971	0.963	0.058	0.035
3 factors	1364.860	87	15.69	0.903	0.883	0.104	0.053
2 factors	4650.904	89	52.26	0.654	0.592	0.194	0.120
1 factor	6069.152	90	67.44	0.547	0.471	0.221	0.146

*4-factor model: lockdown social isolation, lockdown job insecurity, lockdown financial insecurity, lockdown stress; 3-factor model: lockdown social isolation, lockdown job insecurity + lockdown financial insecurity, lockdown stress; 2-factor model: lockdown social isolation, lockdown job insecurity + lockdown financial insecurity + lockdown stress; 1-factor model: lockdown social isolation + lockdown job insecurity + lockdown financial insecurity + lockdown stress. Mindfulness, measured with the summed values for the 6 items, was deemed as a manifest variable and was not included in the CFA model. As a rule of thumb, χ^2^/df ≤ 3 (Hair et al., 2010), CFI and TLI values > 0.90 (Bentler, 1990), RMSEA ≤ 0.05 (Kenny et al., 2015), and SRMR ≤ 0.08 (Hu and Bentler, 1999) indicate a good fit between the model and the data.*

### Measurement Model Assessment

We assessed the measurement model in terms of construct reliability, convergent and discriminant validity. Note that mindfulness is excluded from the measurement model since we followed Feldman et al.’s (2007) suggestions to assess mindfulness by summing up the values for 6 items, and mindfulness is thus treated as a manifest variable.

As shown in Table A1, for each of the constructs, the composite reliability ranges from 0.829 to 0.919, the Cronbach’s alpha varies from 0.824 to 0.918, which are both above the 0.70 threshold, indicating the consistency of the entire scale for each study construct (Hair et al., 2010). We examined average variance extracted (AVE) for convergent validity (cf. Hair et al., 2010; Lim et al., 2022). AVE for each construct varies from 0.621 and to 0.702, herewith all exceeding the 0.50 threshold (Fornell and Larker, 1981), and thus indicating adequate evidence of convergent validity.

Following Hair et al. (2010), we conducted a series of Confirmatory Factor Analyses (CFA) to examine whether the variables were distinct. As shown in Table 2, the proposed four-factor structure (i.e., lockdown social isolation, lockdown job insecurity, lockdown financial insecurity, and lockdown stress) turned out to have the best fit with the data, χ^2^/*df* = 678.974/84 = 5.61, *p* < 0.001, CFI = 0.971, TLI = 0.963, RMSEA = 0.058, SRMR = 0.035, which indicated that the discriminant validity of our study variables was good.

We also followed Podsakoff et al. (2003) to deal with the problem of common-method variance (CMV). Firstly, we used well-validated scales to optimize the psychometric qualities of our measurements. Secondly, we confirmed that all respondents completed the questionnaires anonymously. Finally, Harman’s single-factor test was conducted to examine the CMV, with a series of CFAs in which all the measurement items were loaded onto one common factor (Mossholder et al., 1998). The single-factor model had a very poor fit with the data, χ^2^/*df* = 6069.152/90 = 67.44, *p* < 0.001, CFI = 0.547, TLI = 0.471, RMSEA = 0.221, SRMR = 0.146, which indicated that CMV was not a serious problem in our study.

### Hypotheses’ Testing

**FIGURE 2:**
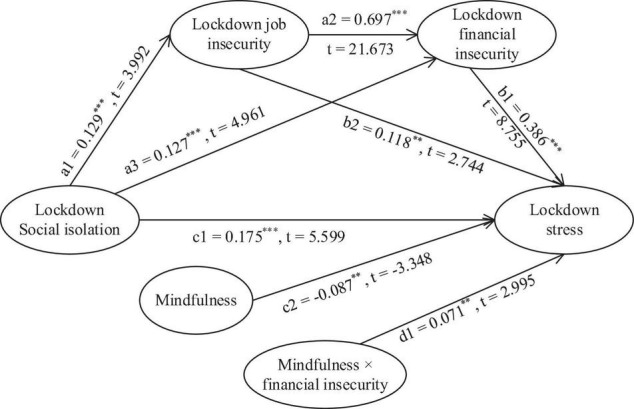
A graphical illustration of parameter estimates. [*p < 0.05, **p < 0.01, and ***p < 0.001. Number of free parameters = 60, Loglikelihood (LL) = −25741.958, AIC = 51603.916, N = 1356. Note: When using the LMS method, Mplus does not produce indices of model fit like CFI and TLI, but produces LL and AIC].

The LMS method (Latent Moderated Structural modeling, cf. Klein and Moosbrugger, 2000) was employed within Mplus 8.3 to test the structural model (see Figure 2), which incorporates all our proposed hypotheses. We firstly estimated a baseline model (the direct effects and the mediation effects included only, i.e., the interaction effect was excluded), which demonstrated a sufficient model fit, χ^2^/*df* = 944.096/196 = 4.82, *p* < 0.001, CFI = 0.945, TLI = 0.937, RMSEA = 0.053, SRMR = 0.058. Subsequently, we added the interaction term to the baseline model, to estimate the proposed model, which significantly improved the model fit, –2ΔLL = 8.996, Δ*df* = 1, *p* < 0.01 [–2ΔLL = –2(LL0-LL1), and is deemed to be chi-square distributed (cf. Gerhard et al., 2015), where LL0 is the log-likelihood of the baseline model, and LL1 is the log-likelihood of the proposed model].

Hypothesis 1 predicted that during the COVID-19 lockdown period, lockdown social isolation was positively associated with employees’ perceived lockdown stress. As shown in Figure 2, the results indicated that after taking the impact of all control variables into account, lockdown social isolation significantly affected lockdown stress (c1 = 0.175, *p* < 0.001). Therefore, Hypothesis 1 was supported with our data.

Hypothesis 2 predicted that lockdown job insecurity partially mediated the relationship between lockdown social isolation to lockdown financial insecurity during the COVID-19 lockdown period. The results as presented in Figure 2 displayed that when the impact of all control variables was taken into account, lockdown social isolation positively affected lockdown job insecurity (a1 = 0.129, *p* < 0.001), lockdown job insecurity positively affected lockdown financial insecurity (a2 = 0.697, *p* < 0.001), and that lockdown social isolation had an indirect effect on lockdown financial insecurity through lockdown job insecurity (a1 × a2 = 0.090, *p* < 0.001). Meanwhile, lockdown social isolation had a positively direct effect on lockdown financial insecurity (a3 = 0.127, *p* < 0.001). Thus, Hypothesis 2 was supported as well with our data.

Hypothesis 3a stated that the relationship between lockdown social isolation and lockdown stress during the COVID-19 lockdown period was partially mediated through lockdown job insecurity. Hypothesis 3b also predicted simple partial mediation through lockdown financial insecurity, and Hypothesis 3c suggested an alternative mediating pathway, where lockdown job insecurity and lockdown financial insecurity sequentially played a mediator role. As shown in Figure 2, lockdown social isolation had a positively direct effect on lockdown job insecurity (a1 = 0.129, *p* < 0.001), lockdown job insecurity had a positively direct effect on lockdown stress (b2 = 0.118, *p* < 0.01), lockdown social isolation had a positively direct effect on lockdown financial insecurity (a3 = 0.127, *p* < 0.001), lockdown financial insecurity had a positively direct effect on lockdown stress (b1 = 0.386, *p* < 0.001), and the relationship between lockdown social isolation and lockdown stress was mediated through lockdown job insecurity (a1 × b2 = 0.015, *p* < 0.05), through lockdown financial insecurity (a3 × b1 = 0.049, *p* < 0.001), and through job insecurity and financial insecurity sequentially (a1 × a2 × b1 = 0.035, *p* < 0.001). Table 3 demonstrates the direct, indirect, and total effects as well as the effect sizes. Thus, H3a, H3b, and H3c were fully supported with our data as well.

**TABLE 3:** Demonstration of the direct, indirect, and total effects.

Paths	Effects	Effect size %
Lockdown social isolation→lockdown stress	0.175***, *t* = 5.599	64%
Lockdown social isolation→lockdown job insecurity→lockdown stress (a1 × b2)	0.015*, *t* = 2.288	6%
Lockdown social isolation→lockdown financial insecurity→lockdown stress (a3 × b1)	0.049***, *t* = 4.392	18%
Lockdown social isolation→lockdown job insecurity → lockdown financial insecurity→lockdown stress(a1 × a2 × b1)	0.035***, *t* = 3.637	13%
Total indirect effects	0.099***, *t* = 6.093	36%
Total effects	0.274***, *t* = 8.096	100%

*p < 0.05, ***p < 0.001.

Hypothesis 4 predicted that employees’ mindfulness was negatively associated with their perceived lockdown stress during the COVID-19 lockdown period, and Hypothesis 5 further predicted that the positive relationship between lockdown financial insecurity and employees’ perceived lockdown stress in the COVID-19 lockdown period was stronger for employees with higher level of mindfulness than those with lower level of mindfulness. The empirical results as shown in Figure 2 illustrated that mindfulness indeed had a significantly negative impact on lockdown stress (c2 = –0.087, *p* < 0.01), and that the interaction term Mindfulness × lockdown financial insecurity had a significantly positive effect on lockdown stress (d1 = 0.071, *p* < 0.01). So, both Hypotheses 4 and 5 were supported with our data as well.

**FIGURE 3:**
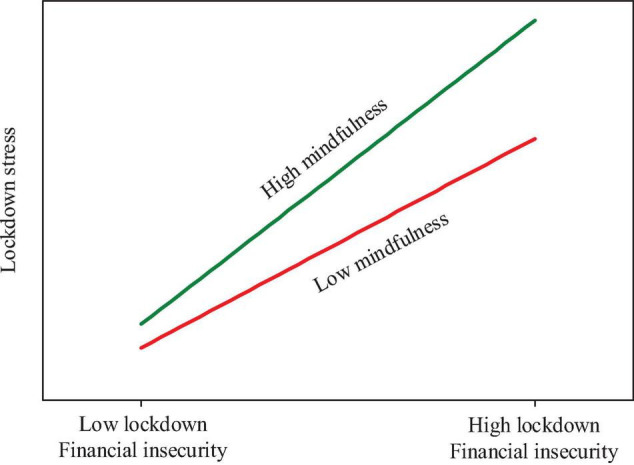
A graphical illustration of moderation by mindfulness.

As regards the outcomes relating to Hypothesis 5, we further assessed the pattern of the moderation effect (Stride et al., 2015) of mindfulness on the relationship between lockdown financial insecurity and lockdown stress (see Figure 3). At low levels of mindfulness (1 SD below the standardized mean), the regression line was tilted to the lower right, indicating lower levels of lockdown stress; at high levels of mindfulness (1 SD above the standardized mean), the regression line was tilted to the higher right, indicating higher levels of lockdown stress. Thus, the pattern of the moderation effect was in line with our expectations (see Figure 3).

## Discussion

### Conclusion and Contributions

Building on COR theory (Hobfoll, 1989, 2001), Psychological Contract theory (Ashford et al., 1989; Keim et al., 2014), Mindfulness theory (Kabat-Zinn, 2015), and Kernis and Goldman’s (2004) Awareness notion, we examined a moderated mediation model with SEM to explore the relationships between lockdown social isolation, lockdown job insecurity, lockdown financial insecurity, lockdown stress, and mindfulness. From the empirical support in our analysis, we can conclude that (1) Lockdown social isolation will increase lockdown stress, both directly and indirectly. The direct effect is considerable (about 60% effect size, see Table 3), and the indirect effect through lockdown job insecurity and lockdown financial insecurity is also substantial (about 40% effect size, see Table 3); (2) Mindfulness will directly decrease lockdown stress. Though the impact coefficient is small, the impact is significant (c2 = –0.087, *p* = 0.001) and important; we believe that anything that can alleviate lockdown stress should be appreciated in the COVID–19 pandemic; (3) However, mindfulness is not always good for lockdown stress alleviation; with the increase of mindfulness, the positive effect of lockdown financial insecurity on lockdown stress is strengthened as well.

To the best of our knowledge, this is the first empirical research using a nation-wide Chinese sample to investigate the relationships between the distinguished model variables among self-quarantined respondents in the COVID-19 pandemic period. In summary, this study contributes to the extant literature in three ways.

Firstly, unlike some researchers who considered the fear of COVID-19 and the COVID event as antecedents of job insecurity (Chen and Eyoun, 2021; Lin et al., 2021), drawing on COR theory (Hobfoll, 1989, 2001), we viewed lockdown social isolation as a predictor in our research model, which offers a psychological perspective to understand and capture the impact of the COVID-19 pandemic on lockdown job insecurity and its consequences. Just as Cai et al. (2021) pointed out that the lockdown policy could reduce the likelihood of returning to work, we suggested that it was lockdown social isolation that resulted in job insecurity by isolating employees at home. Correspondingly, given the lack of research on studying the antecedents of job insecurity (Lee et al., 2018; Lin et al., 2021), our empirical findings that lockdown job insecurity plays a partial mediation role between lockdown social isolation and lockdown stress extend previous academic work, and add to the knowledge in this field using a Chinese sample.

Secondly, through examining the role of lockdown social isolation as an antecedent in our proposed mediation model, with lockdown job insecurity and lockdown financial insecurity as mediators, and with lockdown stress as the response variable, we addressed the impact mechanism through which lockdown social isolation effects individuals’ lockdown stress. In doing so, we extend previous scholarly research which concentrated on the influence of social isolation on job insecurity, financial insecurity, and stress (Brooks et al., 2020; Aguiar-Quintana et al., 2021). Building on COR theory (Hobfoll, 1989, 2001) and Psychological Contract theory (Ashford et al., 1989; Keim et al., 2014), we found that the relationship between lockdown social isolation and lockdown stress is sequentially mediated through lockdown job insecurity and financial insecurity, respectively. More specifically, lockdown social isolation can increase lockdown stress in both direct and indirect ways, that is through the latter mentioned mediators as well.

In line with Wilson et al.’s (2020) work that both job insecurity and financial concerns due to the COVID-19 pandemic are positively related to anxiety, we also found that both lockdown job insecurity and lockdown financial insecurity are positively related to lockdown stress (anxiety). However, unlike Wilson et al.’s (2020) finding that financial concerns do not mediate the relationship between job insecurity and anxiety, we found that lockdown financial insecurity does mediate (though partially) the relationship between lockdown job insecurity and lockdown stress. This difference in outcomes may come from the variation in strictness of the lockdown period, as implemented among different countries and regions. In China, the lockdown and social distancing regulations had been implemented very strictly. For example, during the pandemic, each household could only send one person out, every 2 to 3 days, to purchase daily necessities. Such a strict type of isolation might drive people to feel stressed (anxious and worried) about losing their job and work-related financial loss, due to the uncertainty of the lockdown related to COVID-19.

Thirdly, we also found that mindfulness exerts an impact on lockdown stress, in both direct and indirect ways. In particular, mindfulness has a negative direct effect on lockdown stress, on the one hand, and it has a positive moderating impact on the relationship between lockdown financial insecurity and lockdown stress, on the other hand. That is to say, high levels of awareness may indicate potential costs, such as pain and stress (Kernis and Goldman, 2004). Accordingly, this theoretical insight is important, as during the COVID-19 lockdown period high levels of awareness of lockdown financial insecurity can intensify employee lockdown stress. Our findings also indicate that mindfulness, which apparently is valued by many people who believe that it can reduce their stress and pain in actual life (Hülsheger et al., 2013; Kabat-Zinn, 2015; Dillard and Meier, 2021), can also be an important hindering factor as it seems to strengthen the effect of lockdown financial security on perceived lockdown stress during the COVID-19 period.

In order to cope with the COVID-19 pandemic, mindfulness-based approaches have been widely used to mitigate mental health crises since they can facilitate an individual’s acceptance of uncomfortable, difficult, and painful experiences under present-focused awareness and without judgment (Antonova et al., 2021). Recent research has also shown that mindfulness is negatively related to anxiety, depression, and stress to as considerable extent, even under the COVID-19 pandemic background (Belen, 2022; Yalçın et al., 2022). However, from our study we may conclude that mindfulness is a two-bladed sword. Mindfulness may be a simple and effective tool to relieve employees’ lockdown stress, on the one hand. Yet, simultaneously, it can strengthen the effects of lockdown financial insecurity on lockdown stress.

In China, except for a few big companies such as Alibaba, Huawei, and Didi who have begun to train employees using mindfulness-based approaches, employees in most companies have not experienced and benefited from this mindfulness practice. Correspondingly, most Chinese employees lack cognition and understanding of mindfulness. The power of mindfulness lies in the particular practice and application (Kabat-Zinn, 2015). Therefore, if an individual only holds much self-knowledge of mindfulness without sound practicing, he or she may experience negative effects because a high level of awareness implies potential costs such as pain and stress (Kernis and Goldman, 2004). Hence, for Chinese employees, it seems that the more aware they felt during their lockdown social isolation and financial insecurity, the more stress and pain they experienced during the COVID-19 pandemic.

### Limitations and Future Research Suggestions

Although the findings of this study can serve as a helpful baseline for future research on the relationships between social isolation, job insecurity, financial insecurity, stress, and mindfulness, the present study has some limitations. Firstly, the data set used to examine our research model was collected from respondents’ self-reporting after the full lockdown was canceled, and this may have resulted in some memory and selection biases. Additionally, one might have some concerns about whether common-method bias (Podsakoff et al., 2003) might have affected our findings, though this was not a big problem in this study (ibid.) as the Harman’s single factor test indicated. Besides, the AVE value of mindfulness, which we measured following Feldman et al.’s (2007) recommendations, was relatively low (0.381) in this study, which indicates that an alternative and proper Chinese mindfulness scale should be developed in future scholarly work.

Second, although mindfulness practice plays an important role in improving employees’ mental health, most Chinese employees are still ignorant about it. Accordingly, in this study, we could not divide participants into two groups of employees differentiating between mindfulness practice-experienced individuals versus inexperienced ones. However, as the power of mindfulness lies in its specific practice and application (Kabat-Zinn, 2015), in order to have a more in-depth understanding of the effect of mindfulness on stress, further research is needed wherein the outcomes for experienced and –inexperienced participants are carefully compared. Note that we followed the mindfulness definition by Brown and Ryan (2003, p. 822) because we think that this definition demonstrates the most essential meaning of mindfulness. Specifically, the measure of mindfulness in this study focuses on the dimensions of Attention and Awareness which were drawn from CAMS-R by Feldman et al. (2007). The systematic review by Park et al. (2013) identified numerous definitions and scales measuring mindfulness, but found that none of the scales had sufficient evidence of content validity. So, it will be interesting to apply other definitions and measures of mindfulness to test our model in future research approaches.

Third, the findings of this manuscript are limited due to the cross-sectional design, wherein the sample was recruited in a specific phase of COVID-19 outbreak, and due to the number of items used to calculate the job insecurity score. Moreover, in case the research team members used WeChat to collect data, only those respondents who had a good relationship with these members were selected. Future efforts are needed to avoid these limitations.

Further scholarly work is needed to examine our hypothesized model using a longitudinal approach. Ren et al. (2018), in their meta-analysis, found that although mindfulness-based practices had high immediate effects on alleviating anxiety, these effects did not last. Mindfulness is an individual internal resource (Ni et al., 2021), which may vary across the specific mindfulness-based practices one carries out. As such, follow-up research should collect multi-wave data rather than cross-sectional data to better understand whether mindfulness can generate persistent effects.

Additionally, although the Chinese society is family oriented and Chinese people usually would attach priority to the relationship with their family rather than with their friends, it cannot be denied that some people might put friends or other social relationships above their family in terms of importance. So, it is desirable to include these factors as well, over and above family companion, in future research models.

### Practical Implications

First, in light of the harmful effects of lockdown social isolation related to the COVID-19 pandemic, organizations can take some measures to reduce the extent to which employees perceive themselves to be isolated. For instance, organizations can keep regular online social connections with employees to alleviate this strain.

Second, our findings show that higher perceptions of lockdown job insecurity and financial insecurity result in higher lockdown stress. These outcomes point to the importance of mitigating employees being focused on lockdown job insecurity and financial insecurity in order to avoid their negative psychological reactions. In order to do so, organizations can set up a clear regulation (e.g., organizational care and no layoff plan) to help employees to alleviate the amount of experienced lockdown job insecurity and financial insecurity. Additionally, encouraging employees to work at home or somewhere else, other than at the traditional/formal workplace (that is, introducing a “customized workplace program” during a crisis such as COVID-19), may also contribute to alleviating the worries about financial insecurity, as employees will have the feeling that it is accepted to work in this way without fearing the loss of their job.

Third, our findings suggest that mindfulness can attenuate lockdown stress. Thus, mindfulness-based practices (e.g., meditation, yoga, and mindfulness classes) can be provided by organizations to help employees buffer the negative impact of lockdown stress. At the same time, given that higher levels of mindfulness may evoke a more pernicious impact of lockdown social isolation on lockdown stress, via its interaction with perceived financial insecurity, it is all important that socially isolated persons prevent themselves from an overmuch engagement with mindfulness-based practices during a period of crisis. That is to say, people should prescribe themselves an appropriate or limited amount of time for mindfulness-based practices every day, and they should find helpful substitutes like reading, exercising, playing games with family members, even in very hard times (e.g., during self-quarantine), to avoid preoccupation with the circumstances.

## Data Availability Statement

The data supporting the conclusions of this article are available on request from the authors, though not publicly available due to privacy or ethical considerations.

## Ethics Statement

Ethical review and approval was not required for the study on human participants in accordance with the local legislation and institutional requirements. The patients/participants provided their written informed consent to participate in this study.

## Author Contributions

LZ carried out the data curation and formal analysis. HT, SL, LZ, and ZG carried out the funding acquisition. LZ, SL, and JL investigated the data. LZ and JL performed the methodology. LZ and ZG carried out the project administration. All authors contributed to conceptualization, writing—original draft and review and editing, read, and agreed to the published version of the manuscript.

## Conflict of Interest

The authors declare that the research was conducted in the absence of any commercial or financial relationships that could be construed as a potential conflict of interest.

## Publisher’s Note

All claims expressed in this article are solely those of the authors and do not necessarily represent those of their affiliated organizations, or those of the publisher, the editors and the reviewers. Any product that may be evaluated in this article, or claim that may be made by its manufacturer, is not guaranteed or endorsed by the publisher.

## Footnotes

1 [Welfare] Care for yourself and care for “heart” health ∼ The 8-week course of mindfulness cognitive therapy eMBCT is waiting for you to sign up! https://www.thepaper.cn/newsDetail_forward_7344530, accessed June 18, 2021.

## Appendix

**TABLE A1:** Description of measurement scales.

Scale items (1 = strongly disagree, 5 = strongly agree)	Loadings	Sources of references
***Lockdown social isolation* (CR = 0.829, α = 0.824, AVE = 0.621)**		
I hadn’t seen many of my family members whom I should have seen if there had been no lockdown.	0.554	Ellison and George, 1994; Brummett et al., 2001; Thoits and Hewitt, 2001; Benjamins, 2004; Cornwell and Waite, 2009; Zavaleta et al., 2017
I hadn’t seen many of my relatives whom I should have seen if there had been no lockdown.	0.783	
I hadn’t seen many of my friends whom I should have seen if there had been no lockdown.	0.818	
I hadn’t participated many activities which I should have been in if there had been no lockdown.	0.615	

***Lockdown job insecurity* (CR = 0.866, α = 0.862, AVE = 0.684)**		
During the COVID-19 lockdown,		Anderson and Pontusson, 2007; Carr and Chung, 2014; Vander Elst et al., 2014
I often felt that my workplace might reduce the staff due to business lockdown.	0.775	
I often felt that I might be laid off due to business lockdown.	0.887	
#I couldn’t stop thinking of the difficulty of finding a new job.	–	
I often felt that my current job might become unstable due to the pandemic.	0.814	

***Lockdown financial insecurity* (CR = 0.904, α = 0.903, AVE = 0.702)**		
During the COVID-19 lockdown, I worried that:		Lawrence et al., 2013; Odle-Dusseau et al., 2018; Weinstein and Stone, 2018
the sources of my income would be reduced due to the pandemic.	0.861	
my salary would be lowered.	0.848	
my monthly income would not be able to meet my monthly expenses.	0.701	
my or my family’s total income would go down.	0.852	
the financial situation of me or my family would become (more) difficult.	0.764	

***Lockdown stress* (CR = 0.919, α = 0.918, AVE = 0.695)**		
During the COVID-19 lockdown,		Antony et al., 1998; Lee et al., 2019
I often felt nervous and anxious.	0.856	
I tended to over-react to situations.	0.864	
I felt I was rather touchy.	0.772	
I found it difficult to relax.	0.870	
I found it hard to wind down.	0.798	

**Mindfulness: awareness and attention (CR = 0.764, α = 0.757, AVE = 0.381)**		
I can usually describe how I feel at the moment in considerable detail.	0.500	Feldman et al., 2007; Chan et al., 2016
It’s easy for me to keep track of my thoughts and feelings.	0.382	
I try to notice my thoughts without judging them.	0.610	
It is easy for me to concentrate on what I am doing.	0.636	
I am easily distracted. (Reverse-scored).	0.630	
I am able to pay close attention to one thing for a long period of time.	0.437	

*CR, α, and AVE refer to Composite Reliability, Cronbach’s alpha, and Average Variance Extracted, usually with a threshold of 0.70, 0.70, and 0.50, respectively (cf. Fornell and Larker, 1981; Hair et al., 2010). The values of CR, α, and AVE were calculated within the R package “Lavaan” (Rosseel, 2012) and “SEMTools” (Jorgensen et al., 2021). We followed Feldman et al.’s (2007) suggestions to assess mindfulness by summing up the values for 6 items. # This item was excluded as a result of the scale purification procedure. The item parceling method was also applied in the subsequent analyses.*
